# Multifaceted Excited State Dynamics of Coumarin Dyes Anchored on Al_2_O_3_ Film

**DOI:** 10.3390/molecules28010111

**Published:** 2022-12-23

**Authors:** Hyun Seok Lee, Yun Jeong Na, Chul Hoon Kim, Jae Yoon Shin

**Affiliations:** Department of Advanced Materials Chemistry, Korea University, Sejong 30019, Republic of Korea

**Keywords:** coumarin, time-resolved fluorescence, aggregates, Kasha’s rule, excimer

## Abstract

The co-facially stacked dyes on semiconductor films serve as an alternative model to elucidate the photo-driven exciton dynamics occurring in a molecular assembly. In this study, we report the unique emission properties of coumarin dye adsorbed on the surface of the semiconductor film, measured by ultrafast time-resolved fluorescence. When a rigid coumarin derivative, 7-hydroxycoumarin-3-carboxylic acid (OHCCA), is anchored on the Al_2_O_3_ film, the dye manifests dual emissions from the two lowest excited states. Various anchoring modes of a carboxylic acid group on the Al_2_O_3_ surface are invoked to account for the unusual emission process. Additionally, we identified characteristic transition dipole interactions in the well-stacked dye aggregates, which leads to discernible excitonic splitting in the electronic transitions. Femtosecond time-resolved fluorescence reveals that the excimer formation in the aggregate occurs with the time constant of 550 fs. Picosecond time-resolved emission spectra confirm the subsequent structural relaxations of the nascent excimer. The enhanced transition dipole via the electronic coupling between OHCCA and metal oxide can be responsible for the dual emission and the ultrafast excimer formation.

## 1. Introduction

Exploring the chemical reaction dynamics of a molecular assembly is one of the major subjects of organic photovoltaics [1] and artificial photosynthetic systems [2], where efficient charge transport is essential for the high yield of solar energy conversion. Molecular assemblies such as metal-organic frameworks (MOF) and π-stacked planar perylene derivatives have been widely used as a model for developing such systems [3,4]. Recent studies on these molecular assemblies have shown that photo-driven charge separations in the assembly can be promoted via a slip-stacked geometry [5], indicating that the nature of intermolecular interactions governs the dynamics of Frenkel excitons. On the other hand, in a dye-sensitized solar cell (DSSC) system, photosensitizing dyes are covalently adsorbed on the surface of the semiconductor nanocrystal [6], and they can experience co-facial stacking interactions to form dye aggregates. The molecular aggregates in the DSSC are commonly considered unwanted species because they induce fast non-radiative transitions that can compete with the interfacial charge injection process [7,8]. Developing efficient DSSC systems requires a more comprehensive understanding of the dynamic properties of molecular aggregates on semiconductor nanocrystals. However, ultrafast spectroscopic studies on a single monolayer formed on the semiconductor thin film are still rare.

**Scheme 1:**
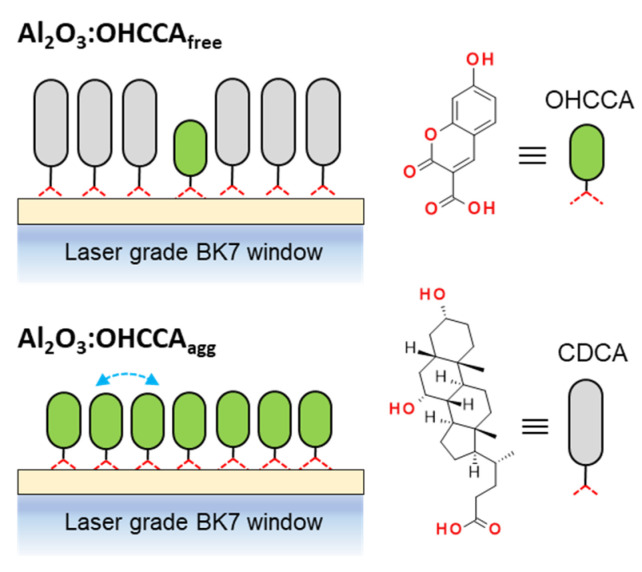
Schematic structures of OHCCA adsorbed on Al_2_O_3_ thin films with (top) and without (bottom) CDCA co-adsorbent.

In this study, we investigated the static and dynamic properties of coumarin dyes anchored on the Al_2_O_3_ film by steady-state and time-resolved fluorescence (TRF) spectroscopies. A rigid coumarin derivative with a carboxylic acid group, 7-hydroxycoumarin-3-carboxylic acid (OHCCA), can form co-facially stacked aggregates on the Al_2_O_3_ film. The rigid structure allows us to focus on intermolecular reactions such as excimer formation without any perturbations from intrinsic structural reorganizations of the monomer. Moreover, the Al_2_O_3_ semiconductor has a high bandgap to avoid any interfacial charge injections into its conduction band. Scheme 1 shows two film samples where OHCCA is adsorbed on the surface of the Al_2_O_3_ thin film with and without co-adsorbent chenodeoxylcholic acid (CDCA). First, to elucidate the intrinsic photophysical properties of an isolated coumarin dye, the excess amount of CDCA (>1:1000 molar ratio) was co-adsorbed on the Al_2_O_3_ film (**Al_2_O_3_:OHCCA_free_**) in which OHCCA could be free from H-type aggregation. For comparison, the stacked OHCCA on the Al_2_O_3_ film was also prepared without CDCA (**Al_2_O_3_:OHCCA_agg_**), from which aggregation-induced dynamics such as excimer formation could be explored exclusively. Note that all the experiments were carried out for the Al_2_O_3_ thin films on the optical window, not colloidal Al_2_O_3_ particles dispersed in the solution, and were free from any contamination by dyes detached from the semiconductor. In addition, the time resolution of TRF measurements was up to 100 fs to address the photo-induced reaction dynamics of the aggregates and probe the dynamic evolutionary processes occurring in the excited state exclusively.

## 2. Results and Discussion

**Figure 1:**
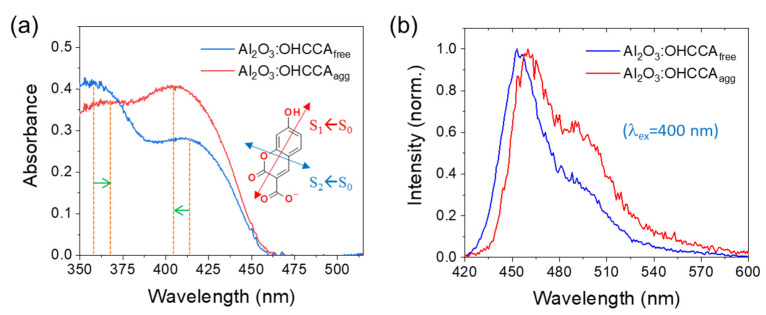
Steady-state measurements: (**a**) Steady-state absorption and (**b**) Steady-state fluorescence spectra of the film samples. The dashed vertical lines in (**a**) indicate the center wavelengths for the two lowest electronic transitions. The inset of (**a**) shows the transition dipole alignments for the S_1_–S_0_ and S_2_–S_0_ transitions. For the fluorescence measurements, the excitation wavelength was 400 nm.

Figure 1 shows the steady-state absorption and fluorescence spectra of **Al_2_O_3_:OHCCA_free_** and **Al_2_O_3_:OHCCA_agg_** films. In the absorption spectrum of **Al_2_O_3_:OHCCA_free_**, the lowest energy transition band near 420 nm appears only when OHCCA is covalently adsorbed on Al_2_O_3_ film (see Figure S1 for the non-binding case). This means that the chemisorption induces a strong electronic delocalization along the binding structure between the dye and metal oxide. The transition band shows a hypsochromic shift to 410 nm in **Al_2_O_3_:OHCCA_agg_** as the number density of the dyes increases. The well-stacked geometry of the dyes on the film enhances the band’s blue shoulder which corresponds to the dipole-allowed transition for H-type aggregates. On the contrary, the higher energy absorption band at around 360 nm exhibits a bathochromic shift in the aggregates. Note that the direction of the transition dipoles for the S_1_←S_0_ absorption band is parallel to the long axis of the coumarin backbone, whereas it is rather perpendicular to the principal axis for the S_2_←S_0_ absorption band (see Figure S2 for the quantum calculation). This may indicate that the origin of the absorption at 360 nm is the S_2_←S_0_ transition band and J-type interactions between the dyes are favorable in the film.

Figure 1b shows the steady-state fluorescence spectra of the films where the excitation wavelength at 400 nm could populate the lowest excited state exclusively (*vide infra*). Compared to **Al_2_O_3_:OHCCA_free_**, the well-stacked OHCCA in **Al_2_O_3_:OHCCA_agg_** exhibits further Stokes shift with a noticeable second vibronic band at 500 nm, indicating a hint of the excimer formation in the aggregates. It is well known that the π–π stacked geometry of dyes in a confined space can induce prominent vibronic features with a large displacement [9,10]. Hence, the well-stacked dyes with vertically oriented transition dipoles can cause an efficient excimer formation with the characteristic fluorescence spectrum.

**Figure 2:**
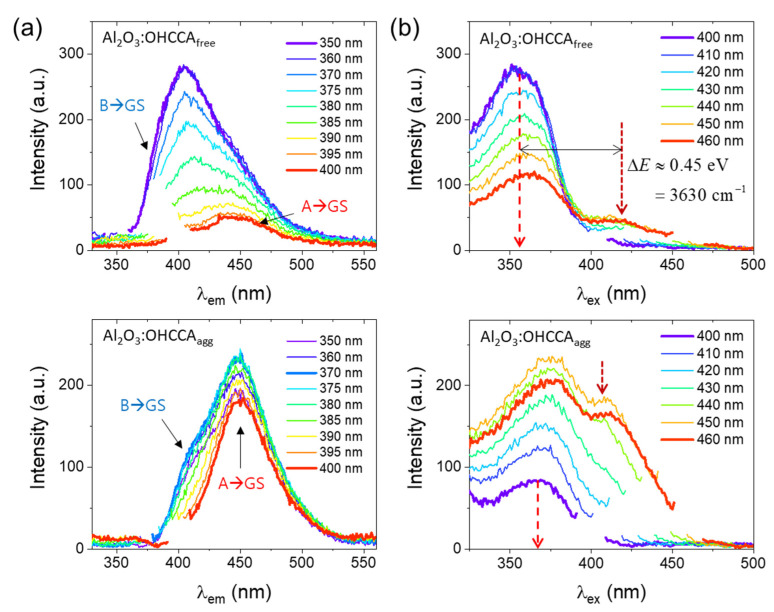
Steady-state (**a**) fluorescence emission spectra at selected excitation wavelengths (λ_ex_) and (**b**) fluorescence excitation spectra at selected emission wavelengths (λ_em_) for **Al_2_O_3_:OHCCA_free_** (top) and **Al_2_O_3_:OHCCA_agg_** (bottom), which were taken from the fluorescence-excitation 2D image (see Figure S3 for the raw image).

The ambiguity in the assignment of the absorption at 360 nm led us to measure the steady-state two-dimensional (2D) fluorescence spectra. The OHCCA films showed unusual behaviors in radiative relaxations from their excited states. When the excitation wavelength (λ_ex_) varied in the range of 350~400 nm, two emission bands appeared near 400 and 450 nm in both film samples (Figure 2a). The higher energy band gradually grew as λ_ex_ became shorter, while the emission band at 450 nm, which corresponds to the lowest electronic transition (A→GS), was dominant at λ_ex_ > 400 nm. The emission band near 400 nm (B→GS) was not attributable to non-binding OHCCA dyes that do not interact with the Al_2_O_3_ surface because a solvent wash removed the non-binding dyes on the films in the sample preparation. The apparent absorption band at 420 nm also confirmed that the OHCCA dyes on the films interact with the Al_2_O_3_ surface (Figure 1a). Therefore, the emission band abnormally observed near 400 nm may be related to the S_2_→S_0_ fluorescence, implying a breakdown of Kasha’s rule. It is well known that the azulene molecule has an exceptionally large energy gap of about 1.7 eV (~13,700 cm^−1^), and so the internal conversion becomes slow because of the poor Franck–Condon factor [11]. For **Al_2_O_3_:OHCCA_free_**, however, the estimated energy gap between the two electronic states was about 0.45 eV (3630 cm^−1^) (see Figure 2b, top), indicating that the gap was not large enough to induce the dual emission involving the S_2_→S_0_ fluorescence. In fact, the azulene derivative with an energy gap of about 4300 cm^−1^ showed only the S_1_→S_0_ fluorescence at room temperature [12]. Therefore, it is reasonable to conclude that Kasha’s rule is still valid in both **Al_2_O_3_:OHCCA_free_** and **Al_2_O_3_:OHCCA_agg_** films.

The fluorescence excitation spectra monitored at λ_em_ = 400 nm revealed a well-separated B←GS transition band at 360 nm (Figure 2b). As λ_em_ became longer, an additional band corresponding to the A←GS transition appeared near 420 nm in both film samples. Compared to **Al_2_O_3_:OHCCA_free_**, the excitation spectrum of **Al_2_O_3_:OHCCA_agg_** monitored at λ_em_ = 460 nm exhibited a dramatically intensified and slightly blue-shifted A←GS transition band with the B←GS transition band being red-shifted (see vertical dashed lines in Figure 2b). This is consistent with the absorption measurements shown in Figure 1a. The enhanced A←GS transition is mainly due to the effective H-type interaction on the film, thereby increasing the oscillator strength for the dipole-allowed transition.

On the other hand, the shoulder of emission at 400 nm (B→GS) was not attributed to the S_2_-S_0_ transition but may be due to the excitonic splitting of the lowest excited state (S_1_). Note that two excitation bands at 360 and 420 nm show the opposite shifting behaviors when OHCCA forms the aggregate on the surface of Al_2_O_3_ (see Figure 2b). In the aggregates, one can attribute the H-type excitonic coupling to account for the hypsochromic shift of the lowest absorption band at 420 nm. However, the opposite behavior of the band at 360 nm is still counterintuitive. Instead, we assume the other possibilities on the origin of the B→GS transition: (1) the impurity of the OHCCA molecule and (2) structural heterogeneity via the various adsorption modes of a carboxylic acid group.

M. Z. Shafikov et al. reported that the origin of the dual emission of a coumarin derivative (C-2) in the solution is the impurity that is one of the synthetic intermediates [13]. The authors confirmed that C-2 has an energy gap (S_2_-S_1_) of about 4000 cm^−1^, and the purified C-2 does not show any dual fluorescence. Although this is similar to the case of the OHCCA on film, the impurity of OHCCA should be negligible: only the **Al_2_O_3_:OHCCA** films exhibited the dual emission while the drop-casted film (the non-binding dye) did not show any additional band at 450 nm as shown in Figure S1. Y. Gao et al. reported that three anchoring modes of retinoic acid, which contain a carboxylic acid group adsorbed on TiO_2_, reveal different absorption transitions [14]. They showed that the distance between a dye and the surface of the metal oxide after the formation of the H–bond significantly affects the coupling between the dye’s excited state and the conduction band states of the semiconductor. Therefore, it is reasonable to assume that the electronic transitions of OHCCA anchored on the surface of the Al_2_O_3_ film are strongly dependent on the anchoring mode of the carboxylic acid group. Moreover, different anchoring modes of a carboxylic acid group on the surface of Al_2_O_3_ might be responsible for the dual emission and the opposite shifting behaviors of the corresponding absorption bands.

**Figure 3:**
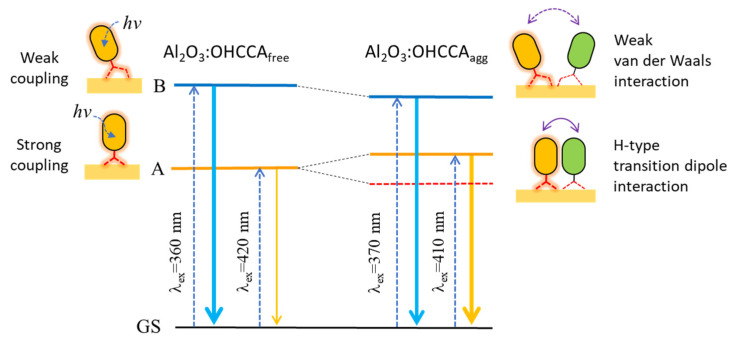
Schematic energy level diagrams for the electronic transitions in the film samples. A, B, and GS denote the bidentate binding state, the monodentate binding state, and the ground state, respectively (see proposed anchoring modes in Figure S4).

The electronic transition pathways in the film samples are summarized in Figure 3. In **Al_2_O_3_:OHCCA_free_**, the bidentate binding mode (A) causes the strongest orbital mixing to give the lowest electronic transition at 420 nm. The monodentate binding mode (B) with/without an H–bond reveals the tilted binding geometry on the surface of Al_2_O_3_, which induces relatively weak orbital mixing to give a higher electronic transition at 360 nm [14]. As a result, the dual emission can be ascribed to the mixture of OHCCA with different anchoring modes. In **Al_2_O_3_:OHCCA_agg_**, the monodentate binding geometry is heterogeneous in terms of the intermolecular orientation, i.e., the co-facial stacking interaction is less probable in randomly oriented geometry. The van der Waals interactions between weakly coupled dyes are expected, and it can stabilize the excited state of OHCCA (B) to give the red-shifted absorption near 360 nm (see Figure 1). Accordingly, only some portion of the aggregate with the bidentate binding one reveals the well-stacked assembly in the film. Influenced partly by the strong orbital coupling between the dye and Al_2_O_3_, the intermolecular interaction between the dyes in **Al_2_O_3_:OHCCA_agg_** is effective enough to induce the excitonic splitting of the electronic transitions.

**Figure 4:**
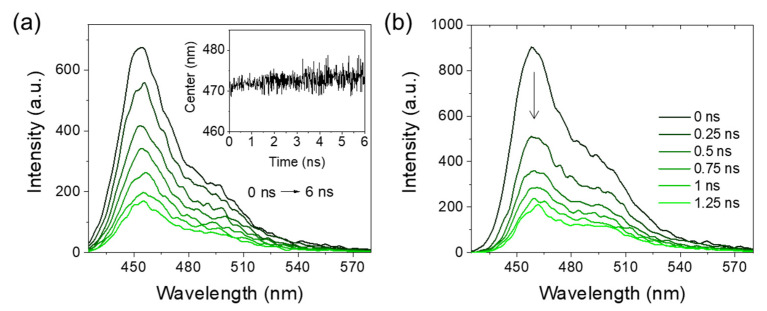
Time-resolved emission spectra (TRES) of (**a**) **Al_2_O_3_:OHCCA_free_** and (**b**) **Al_2_O_3_:OHCCA_agg_**. The inset of (**a**) shows the first moment (center wavelength weighted by fluorescence intensity) of TRES as a function of time. The excitation wavelength was 400 nm.

To elucidate the ultrafast reaction dynamics of the OHCCA aggregates without any perturbations from higher-lying excited states in the energy (B), we focused on the relaxation dynamics of the lowest excited state (A), employing time-resolved fluorescence (TRF) spectroscopy with 400 nm excitation. Figure 4 displays the time-resolved emission spectra (TRES) of the film samples recorded by the time-correlated single photon counting (TCSPC) method with a time resolution of 50 ps (see Figure S5 for the raw images). In **Al_2_O_3_:OHCCA_free_**, TRES slowly decay in intensity over 6 ns and show no dynamic Stokes shift with a large displacement. As shown in the inset of Figure 4a, the first moment of TRES, which represents the center wavelength of the spectrum weighted by fluorescence intensity, does not change much for up to 6 ns. By contrast, the TRES of **Al_2_O_3_:OHCCA_agg_** decays relatively fast during 1 ns with a gradual growth of the red shoulder at 500 nm (Figure 4b).

**Figure 5:**
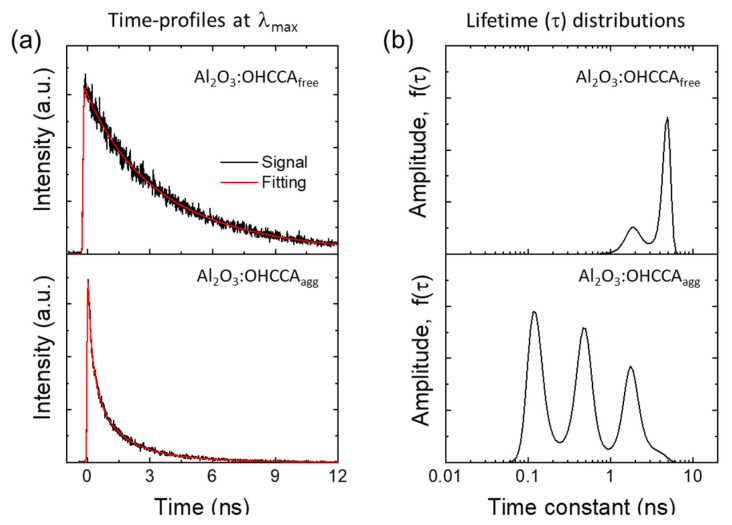
Analysis of time-resolved fluorescence (TRF) of **Al_2_O_3_:OHCCA_free_** (top) and **Al_2_O_3_:OHCCA_agg_** (bottom): (**a**) Time-profiles of TRF at 460 nm with fits (red lines) and (**b**) Lifetime distributions obtained by the maximum entropy method.

The decay profiles of TRF at the maximum wavelength, 460 nm, clearly demonstrate faster decay components in **Al_2_O_3_:OHCCA_agg_** (Figure 5a). For a quantitative comparison, we used the maximum entropy method (MEM) to calculate the lifetime distributions from the fluorescence decay profiles of the film samples, as depicted in Figure 5b. The MEM is commonly used to obtain the distribution of rates (or time constants) in the logarithm scale [15]. In the lifetime distribution of **Al_2_O_3_:OHCCA_free_**, there are two bands in the time constants longer than 1 ns with a dominant one near 5 ns, indicating that isolated OHCCA on the film has a rigid structure and, thus, no fast relaxation processes via structural deformations such as the twisting motion are observed. However, in the lifetime distribution of **Al_2_O_3_:OHCCA_agg_**, two prominent bands appear in the time constants shorter than 1 ns. Note that there are no ultrafast components shorter than 50 ps in the picosecond lifetime distribution. The excimer formation (or charge transfer exciton formation) is known to occur typically within a 1 ps time scale for the well-stacked perylene derivatives in a confined geometry [5,16]. In the aggregated sample, OHCCA dyes can effectively interact with each other in the 2D space to give the excitonic splitting in the absorption spectra, as shown in Figure 1a. However, the time scale of about 100 ps observed in Figure 5b is too long to represent the excimer formation of OHCCA dyes on the film. The femtosecond fluorescence up-conversion measurements could reveal an ultrafast decay with a time constant of 550 fs in the aggregated sample (*vide infra*). That is, we can conclude that the ultrafast decay within 1 ps is drastically attenuated by the convolution-integral with the instrument response function (IRF) of about a 50 ps width, and the observed slow kinetics in the TCSPC experiment is rather related to any subsequent relaxation processes of a nascent excimer.

**Figure 6:**
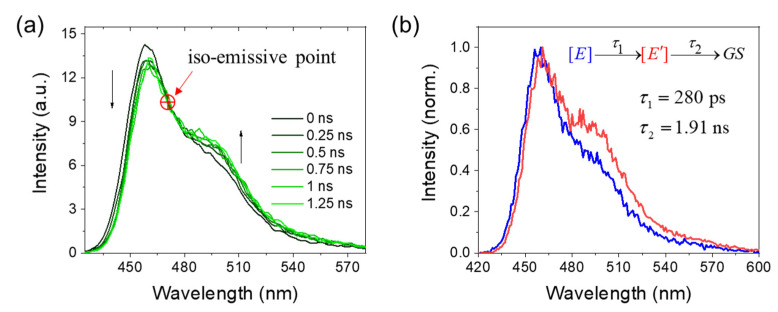
(**a**) Time-resolved area normalized emission spectra (TRANES) of **Al_2_O_3_:OHCCA_agg_** at selected times and (**b**) Evolution associated spectra (EAS) obtained from the global analysis. The inset of (**b**) shows the reaction path and associated time constants. A red open circle with a plus sign in (**a**) indicates the iso-emissive point of TRANES.

To elucidate the origin of the slow relaxation dynamics of **Al_2_O_3_:OHCCA_agg_**, the picosecond TRES were further analyzed by the time-resolved area normalized emission spectra (TRANES). The TRANES is commonly used to identify the characteristic excited state dynamics of a dye without concerning any population decay in the excited state [17]. Figure 6a displays the TRANES of **Al_2_O_3_:OHCCA_agg_**, which has an iso-emissive point near 470 nm. This indicates that two independent emissive states are involved in the relaxation processes [14]. Since an ultrafast decay component shorter than 1 ps cannot be resolved in the picosecond TRES, its contribution to the TRES is negligible. The intermolecular energy transfer occurring in the π-stacked OHCCA on the film may be related to the spectral shift, but we ruled out this possibility because a localization of coherent excitons might be ultrafast in the stacked aggregates [18]. Moreover, the internal conversion from the S_2_ state and the intramolecular vibrational relaxations (IVR) from the vibrationally hot band of the S_1_ state are not responsible here because the excitation at 400 nm can populate the S_1_ state exclusively (see Figure 2), and the IVR process is ultrafast in a condensed phase [19]. Therefore, it is reasonable to conclude that two independent emissive states in TRANES likely correspond to the nascent excimer in the lowest excited state and its subsequently relaxed form, which are observed beyond 100 ps and unrelated to the nascent excimer formation.

The time scale for the subsequent relaxation pathway can be determined by the evolution-associated spectra (EAS) analysis of the TRES image, as shown in Figure 6b. Here, we implemented the first-order serial reaction scheme, E→E’→ground state (GS), in the global target analysis where E and E’ denote the nascent excimer and subsequently relaxed excimer, respectively. The best fit with a simple two-step model results in the time constants of 280 ps and 1.91 ns (see Figure S6 for the detailed results). The shorter time constant (280 ps) represents the time scale of structural relaxation. Note that the MEM only gives a lifetime distribution for the decay profile recorded at a single wavelength so that the redundant lifetime band near 500 ps in Figure 5b can be ascribed to the associated evolutions of two closely lying emissive states, not the actual population change.

**Figure 7:**
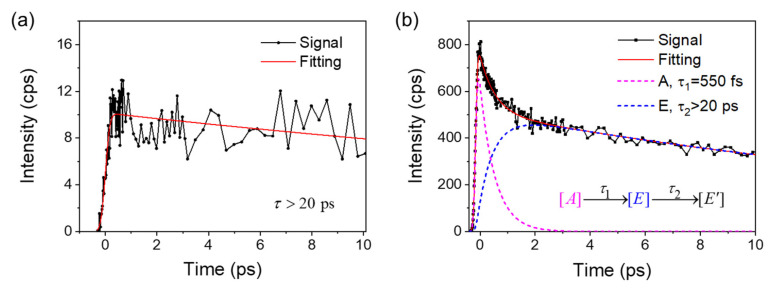
Femtosecond fluorescence up-conversion decay profiles of (**a**) **Al_2_O_3_:OHCCA_free_** and (**b**) **Al_2_O_3_:OHCCA_agg_** with fits (red lines). The detection wavelength was 450 nm. The dashed lines in (**b**) are the population of each species used in the global analysis with the reaction scheme presented in the inset.

For further investigations on ultrafast relaxation processes in the excited state, we measured the fluorescence up-conversion with a time resolution of about 100 fs (Figure 7). It should be noted that the up-conversion signal of **Al_2_O_3_:OHCCA_free_** is very weak, as shown in Figure 7a, due to the extremely low OHCCA concentration on the film. Nonetheless, no fast relaxation shorter than 20 ps was observed in the **Al_2_O_3_:OHCCA_free_** sample, which is consistent with the MEM analysis of the picosecond TRF shown in Figure 5b. This strongly supports the fact that isolated OHCCA on the film undergoes no fast structural relaxations in the excited state and, therefore, shows no discernible dynamic Stokes shift in the picosecond TRES. On the other hand, an ultrafast decay shorter than 1 ps emerges in the up-conversion signal at 450 nm when the OHCCA dyes form H-type aggregates on the film. That is, the origin of the fast decay should be related to population relaxation dynamics occurring in the aggregates. Note that this component is dominant even at 525 nm in the emission band’s red shoulder (see Figure S7). Since the TRES of the excimer is not fully separated in the energy from the TRES of the Franck–Condon state, the decay profiles recorded at a single wavelength can be complicated by the considerable spectral overlap, as depicted in Figure 6b. Again, to quantitatively determine the rate of excimer formation, we employed the first-order reaction scheme, A→E→E’, where the A term denotes the Franck–Condon state of the OHCCA aggregate. As shown in Figure 7b, the single decay profile is well described by the sum of A and E terms. The A term is dominant at both 450 and 525 nm, indicating that the excimer formation causes a sudden change in oscillator strength and reduces the emission intensity over the whole spectral range.

**Figure 8:**
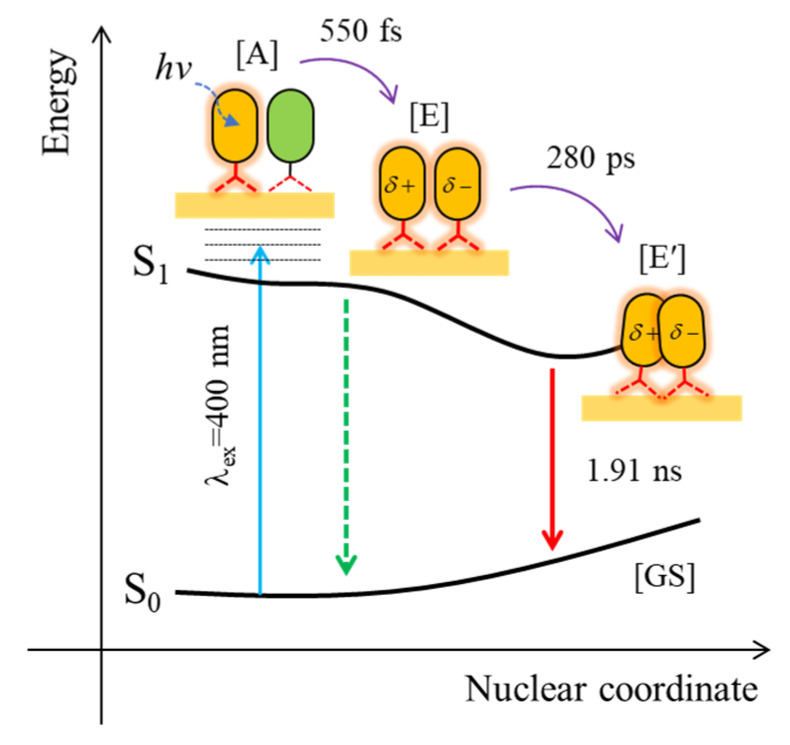
Proposed reaction pathways of **Al_2_O_3_:OHCCA_agg_** film in the excited state.

The overall reaction pathways for the excited state dynamics of **Al_2_O_3_:OHCCA_agg_** are summarized in Figure 8. Upon excitation to the Franck–Condon state at 400 nm, OHCCA in aggregate can immediately undergo excimer formation with a time constant of about 550 fs, as demonstrated by the fluorescence up-conversion experiments. After the nascent excimer formation, subsequent structural relaxations proceed with a time constant of 280 ps. Skeletal motions changing the intermolecular orientation and separation distance may be responsible for this structural reorganization.

## 3. Materials and Methods

### 3.1. Sample Preparation and Absorption Measurement

OHCCA and CDCA were purchased from Sigma-Aldrich (St. Louis, MO, USA) and TCI (Tokyo, Japan), respectively, and were used without further purification. For the preparation of semiconductor film samples, a homemade Al_2_O_3_ paste, which is composed of 1.5 g dispersion of 20 wt % Al_2_O_3_ (Sigma-Aldrich, <50 nm) in isopropanol and an organic binder (4g terpineol and 0.5 g ethyl cellulose mixed in isopropanol), were spin-coated on a BK7 glass substrate (1-inch diameter, 2 mm thickness, WIN-254-020-BK7, Castech Inc., Fuzhou, China) and baked in a muffle furnace. Then, the Al_2_O_3_ films were soaked in a 10 μM solution of OHCCA in ethanol (or OHCCA/CDCA in ethanol at a 1:1000 molar ratio) in the dark for 24 h and were gently washed with ethanol to remove residuals. A home-built absorption measurement setup using an integrated sphere was employed to record steady-state absorption spectra of the opaque films in the range of 350 nm~600 nm.

### 3.2. Time-Resolved Fluorescence

The light source was a home-built cavity-dumped Kerr-lens mode-locked Ti:sapphire oscillator operating at a 500 kHz repetition rate. The 800 nm output of the femtosecond laser was doubled in frequency using a 100 μm thick BBO (β-barium borate) crystal to generate excitation pulses at 400 nm. A parabolic mirror was employed to focus the excitation beam onto the film sample and to collect fluorescence with a confocal geometry. The output was sent to a monochromator (SP-2155, Princeton Instruments, Acton, MA, USA) and detected with a single-photon counting module (id 100-50, ID Quantique, Geneva, Switzerland). A commercial TCSPC board (SPC-130-EMN, Becker & Hickl Inc., Berlin, Germany) was used to record time-resolved fluorescence with a time resolution of about 50 ps. All the instruments were controlled in unison by using a home-built codes written in LabVIEW software (LabVIEW 2016, 16.0, 32 bit, National Instruments, Austin, TX, USA), which allowed us to record the time-resolved emission spectra (TRES) directly.

Noncollinear fluorescence up-conversion methods were employed to record time-resolved fluorescence with a time resolution of about 100 fs [20,21]. For the up-conversion experiment, the same femtosecond light source was used as in the TCSPC, and the second harmonic output at 400 nm was used as the pump, with the residual fundamental pulses serving as the gate. The nonlinear mixing with an external angle of 20° for the fluorescence and the gate pulse was used to minimize the group velocity mismatch in a 500 mm thick BBO crystal. The instrument response of the setup was estimated to be about 100 fs (FWHM) from the cross-correlation measurement between the pump and the gate pulses.

## 4. Conclusions

The effect of chemisorption on the population relaxation dynamics of a coumarin dye was systematically investigated by ultrafast fluorescence spectroscopy. A rigid coumarin derivative, OHCCA, forms the π-stacked aggregates on the semiconductor thin film, which seems comparable to the covalently linked π-stacked perylene derivatives [4]. The transition dipole interaction of the anchored dyes is strong enough to induce the discernible excitonic splitting in the absorption spectrum. For the lowest electronic transition near 400 nm, however, the transition dipole moment is vertically aligned to the long axis of the coumarin backbone, leading to the effective H-type interaction in the aggregate. In addition, the anchored dyes show a dual emission, which is highly influenced by the hindered structural motions of OHCCA and the anchoring mode of the carboxylic acid group on the surface of Al_2_O_3_. Upon 400 nm excitation to the Franck–Condon state of **Al_2_O_3_:OHCCA_agg_**, ultrafast excimer formation occurs with subsequent structural relaxation. The changes in the intermolecular orientation and separation distance are likely responsible for the structural reorganization causing the slow dynamic Stokes shift in the TRES. In this work, the overall photo-induced relaxation processes can be influenced by the strength of chemisorption. In other words, the type of anchoring group and semiconductor can modulate the stacking interaction between the dyes on the film. The excimer formation can be accelerated further with such modifications, serving as a good test bed for studying exciton dynamics in a molecular assembly. The accelerated excimer formation on the semiconductor film can deteriorate the charge injection efficiency because it will severely compete with the ultrafast interfacial charge injection that usually occurs within 1 ps in the TiO_2_ film [22]. Hence, understanding chemisorption’s role is essential to improve the charge injection efficiency of photosensitizing materials adsorbed on semiconductor nanocrystals and the performance of the photovoltaic system.

## Data Availability

Not applicable.

## Supplementary Materials

The following supporting information can be downloaded at: https://www.mdpi.com/article/10.3390/molecules28010111/s1, Figure S1: 2D fluorescence spectra of the drop casted OHCCA/CDCA film; Figure S2: TD-DFT calculation with B3LYP/6-31G(d) basis for the deprotonated OHCCA; Figure S3: 2D fluorescence spectra of **Al_2_O_3_:OHCCA_free_** and **Al_2_O_3_:OHCCA_agg_** films; Figure S4: Two representative anchoring mode of OHCCA on the surface of Al_2_O_3_. Figure S5: Picosecond TRES images of **Al_2_O_3_:OHCCA_free_** and **Al_2_O_3_:OHCCA_agg_** films; Figure S6: The global target analysis of TRES data for **Al_2_O_3_:OHCCA_agg_** film; Figure S7: Model populations and time constants of the first-order kinetic fit to the fluorescence up-conversion signal of **Al_2_O_3_:OHCCA_agg_**. References [23,24,25,26] are cited in supplementary materials.

## Sample Availability

Samples of the compounds are not available from the authors.

## References

[1] Jakowetz A.C., Bohm M.L., Sadhanala A., Huettner S., Rao A., Friend R.H. (2017). Visualizing excitations at buried heterojunctions in organic semiconductor blends. Nat. Mater..

[2] Son H.J., Pac C., Kang S.O. (2021). Inorganometallic Photocatalyst for CO_2_ Reduction. Acc. Chem. Res..

[3] Son H.J., Jin S.Y., Patwardhan S., Wezenberg S.J., Jeong N.C., So M., Wilmer C.E., Sarjeant A.A., Schatz G.C., Snurr R.Q. (2013). Light-Harvesting and Ultrafast Energy Migration in Porphyrin-Based Metal-Organic Frameworks. J. Am. Chem. Soc..

[4] Wurthner F., Saha-Moller C.R., Fimmel B., Ogi S., Leowanawat P., Schmidt D. (2016). Perylene Bisimide Dye Assemblies as Archetype Functional Supramolecular Materials. Chem. Rev..

[5] Kim T., Lin C.J., Schultz J.D., Young R.M., Wasielewski M.R. (2022). π-Stacking-Dependent Vibronic Couplings Drive Excited-State Dynamics in Perylenediimide Assemblies. J. Am. Chem. Soc..

[6] Hagfeldt A., Boschloo G., Sun L.C., Kloo L., Pettersson H. (2010). Dye-Sensitized Solar Cells. Chem. Rev..

[7] Lu H.P., Tsai C.Y., Yen W.N., Hsieh C.P., Lee C.W., Yeh C.Y., Diau E.W.G. (2009). Control of Dye Aggregation and Electron Injection for Highly Efficient Porphyrin Sensitizers Adsorbed on Semiconductor Films with Varying Ratios of Coadsorbate. J. Phys. Chem. C.

[8] Son H.J., Kim C.H., Kim D.W., Jeong N.C., Prasittichai C., Luo L.L., Wu J.S., Farha O.K., Wasielewski M.R., Hupp J.T. (2015). Post-Assembly Atomic Layer Deposition of Ultrathin Metal-Oxide Coatings Enhances the Performance of an Organic Dye-Sensitized Solar Cell by Suppressing Dye Aggregation. ACS Appl. Mater. Interfaces.

[9] Hestand N.J., Spano F.C. (2018). Expanded Theory of H- and J-Molecular Aggregates: The Effects of Vibronic Coupling and Intermolecular Charge Transfer. Chem. Rev..

[10] Gao F., Zhao Y., Liang W.Z. (2011). Vibronic Spectra of Perylene Bisimide Oligomers: Effects of Intermolecular Charge-Transfer Excitation and Conformational Flexibility. J. Phys. Chem. B.

[11] Turro N.J. (1991). Modern Molecular Photochemistry.

[12] Eber G., Grüneis F., Schneider S., Dörr F. (1974). Dual fluorescence emission of azulene derivatives in solution. Chem. Phys. Lett..

[13] Shafikov M.Z., Brandl F., Dick B., Czerwieniec R. (2019). Can Coumarins Break Kasha’s Rule?. J. Phys. Chem. Lett..

[14] Gao Y., Lockart M., Kispert L.D., Bowman M.K. (2019). Photoinduced Charge Separation in Retionic Acid on TiO_2_: Comparision of Three Anchoring Modes. J. Phys. Chem. C.

[15] Kumar A.T.N., Zhu L.Y., Christian J.F., Demidov A.A., Champion P.M. (2001). On the rate distribution analysis of kinetic data using the maximum entropy method: Applications to myoglobin relaxation on the nanosecond and femtosecond timescales. J. Phys. Chem. B.

[16] Lin C.J., Kim T., Schultz J.D., Young R.M., Wasielewski M.R. (2022). Accelerating symmetry-breaking charge separation in a perylenediimide trimer through a vibronically coherent dimer intermediate. Nat. Chem..

[17] Koti A.S.R., Krishna M.M.G., Periasamy N. (2001). Time-resolved area-normalized emission spectroscopy (TRANES): A novel method for confirming emission from two excited states. J. Phys. Chem. A.

[18] Kaufmann C., Kim W., Nowak-Krol A., Hong Y., Kim D., Wurthner F. (2018). Ultrafast Exciton Delocalization, Localization, and Excimer Formation Dynamics in a Highly Defined Perylene Bisimide Quadruple pi-Stack. J. Am. Chem. Soc..

[19] Elsaesser T., Kaiser W. (1991). Vibrational and Vibronic Relaxation of Large Polyatomic Molecules in Liquids. Annu. Rev. Phys. Chem..

[20] Rhee H., Joo T. (2005). Noncollinear phase matching in fluorescence upconversion. Opt. Lett..

[21] Kim C.H., Joo T. (2008). Ultrafast time-resolved fluorescence by two photon absorption excitation. Opt. Express.

[22] Rehm J.M., McLendon G.L., Nagasawa Y., Yoshihara K., Moser J., Gratzel M. (1996). Femtosecond electron-transfer dynamics at a sensitizing dye-semiconductor (TiO_2_) interface. J. Phys. Chem..

[23] van Stokkum I.H.M., Larsen D.S., van Grondelle R. (2004). Global and target analysis of time-resolved spectra. BBA-Bioenergetics.

[24] Henry E.R., Hofrichter J. (1992). Singular Value Decomposition-Application to Analysis of Experimental-Data. Method Enzymol..

[25] Veldkamp B.S., Han W.S., Dyar S.M., Eaton S.W., Ratner M.A., Wasielewski M.R. (2013). Photoinitiated multi-step charge separation and ultrafast charge transfer induced dissociation in a pyridyl-linked photosensitizer-cobaloxime assembly. Energy Environ. Sci..

[26] Frisch M.J., Trucks G.W., Schlegel H.B., Scuseria G.E., Robb M.A., Cheeseman J.R., Scalmani G., Barone V., Petersson G.A., Nakatsuji H. (2016). Gaussian 16W Rev. B.01.

