# Supplementary material for: Multifaceted Excited State Dynamics of Coumarin Dyes Anchored on Al2O3 Film
Source: Molecules. 2022 Dec 23;28(1):111. doi: 10.3390/molecules28010111 (PMC9821847; doi:10.3390/molecules28010111)
Supplement: Supplementary file 1 [file molecules-28-00111-s001.zip › molecules-2069382-supplementary.pdf]

Supporting Information for

Multifaceted Excited State Dynamics of Coumarin Dyes Anchored on Al_2O_3 Films

Hyun Seok Lee, Yun Jeong Na, Chul Hoon Kim*, and Jae Yoon Shin*

Department of Advanced Materials Chemistry, Korea University, Sejong 30019, South Korea

*Email: chulhoon@korea.ac.kr and jaeyoonshin@korea.ac.kr

Nonlinear least squares fitting for femtosecond up-conversion profiles

Femtosecond time-profiles measured by the up-conversion technique were fitted according to the first-order serial reaction schemes shown below.

$$\mathrm{Al_2O_3{:}OHCCA_{free}}:\ A \xrightarrow{k} GS$$
$$\mathrm{Al_2O_3{:}OHCCA_{agg}}:\ A \xrightarrow{k_1} E \xrightarrow{k_2} E'$$

The up-conversion time-profile, $I_{upc}(t)$ can be described by a convolution integral between a model function, $I_{fluo}(t)$ from the reaction scheme and a Gaussian shaped instrument response function, $I_{IRF}(t)$ as follows:

$$I_{upc}(t) = \int_0^\infty I_{fluo}(t) I_{IRF}(t-\tau)\,d\tau\text{, where }\ I_{IRF}(t) = \frac{1}{\sqrt{2\pi}\sigma}\exp\left(-\frac{(t-t_0)^2}{2\sigma^2}\right)$$

Here, t_0 is the time-zero position, and σ is the standard deviation ($\mathrm{FWHM} = 2\sqrt{2\ln 2}\times\sigma$) of the normalized Gaussian function. The population model functions, $I_{fluo}(t)$ are solved from the rate equations for the corresponding reaction schemes as follows.

$$\mathrm{Al_2O_3{:}OHCCA_{free}}:\ I_{fluo}(t) = I_0 \times e^{-kt}$$

$$\mathrm{Al_2O_3{:}OHCCA_{agg}}:\ I_{fluo}(t) = I_{fluo}^A(t) + I_{fluo}^E(t),\quad \begin{cases} I_{fluo}^A(t) = I_{fluo}^A(0)\times \mathrm{e}^{-k_1\cdot t} \\ I_{fluo}^E(t) = I_{fluo}^E(0)\left(\dfrac{k_1}{k_2-k_1}\right)\times\left(\mathrm{e}^{-k_1 t} - e^{-k_2 t}\right) \end{cases}$$

For **Al₂O₃:OHCCA$_{\mathbf{free}}$**, the following analytical solution for the convolution integral of the single exponential decay function was used (see Figure 7a).

$$I_{upc}(t) = \frac{I_0}{2}\times\exp\left[-\frac{t-t_0}{\tau} + \frac{1}{2}\left(\frac{\sigma}{\tau}\right)^2\right]\times\left\{1 + erf\left[\frac{1}{\sqrt{2}}\left(\frac{t-t_0}{\tau}\right) - \frac{\sigma}{\tau}\right]\right\}\text{, where } \tau=1/k.$$

For **Al₂O₃:OHCCA$_{\mathbf{agg}}$**, the model function can be expressed as the sum of three analytical solutions because of three exponential terms. Two up-conversion profiles at 450 nm and 525 nm were fitted globally by sharing two time constants ($\tau_1=1/k_1$, and $\tau_2=1/k_2$) in the model (see Figure 7b and Figure S6).

Global target analysis for picosecond TRES

The TRES (2D image) measured using the TCSPC setup were spectrally deconvoluted by the global target analysis based on the singular value decomposition as described elsewhere. [1-3] A 2D model function, M(λ,t) matrix, can be expressed as

$$M(\lambda,t) = \sum_{k=1}^{2} F(\lambda,k) \times D(k,t)$$

, where F(λ,k) is a 2D matrix for decay (or evolution) associated fluorescence spectra, D(k,t) is a 2D matrix for transient response functions for the corresponding F terms, and k is a singular value index from 1 to 2 (two independent emissive species). The decay profiles, D(k,t), can be described as a convolution integral between the population functions from the first-order serial kinetic scheme, S(k,t) and an instrument response function, I(t).

$$D(k,t) = \int_0^{\infty} S(k,t) \cdot I(t-\tau) d\tau$$

Here, the kinetic scheme, S(k,t) has the form shown below

$$S(1,t) \xrightarrow{k_1 = 1/\tau_1} S(2,t) \xrightarrow{k_2 = 1/\tau_2} GS \quad \Rightarrow \quad \begin{cases} S(1,t) = e^{-k_1 t} \\ S(2,t) = \left(\dfrac{k_1}{k_2 - k_1} \right) \times \left[e^{-k_1 t} - e^{-k_2 t} \right] \end{cases}$$

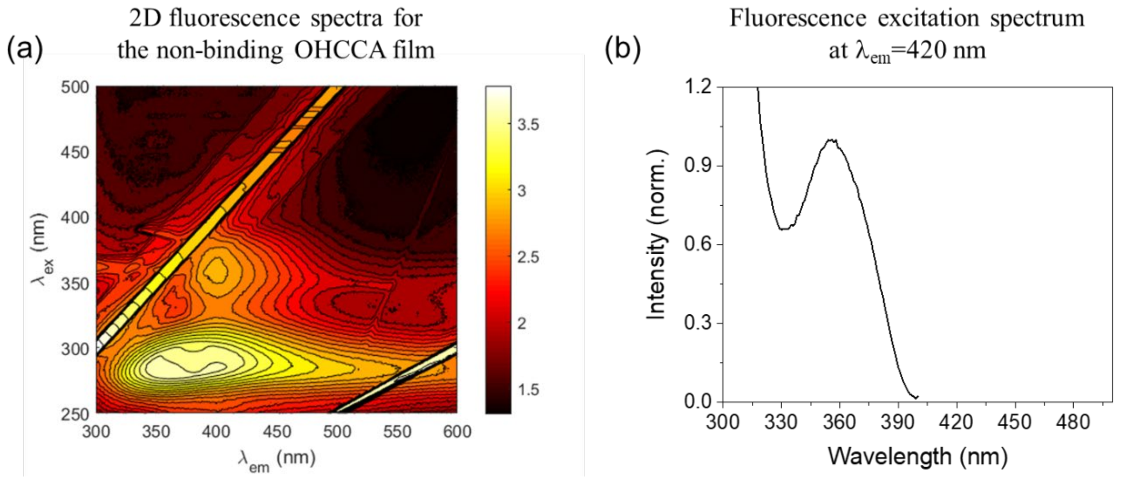

Figure S1. (a) 2D fluorescence spectra of the drop-casted OHCCA/CDCA (1:1000 ratio) film, where the strong emission intensity at the excitation of 290 nm is due to the BK7 window. (b) Fluorescence excitation spectrum at λ_{em}=420 nm, which mimics the absorption spectrum of the non-binding OHCCA on the optical window.

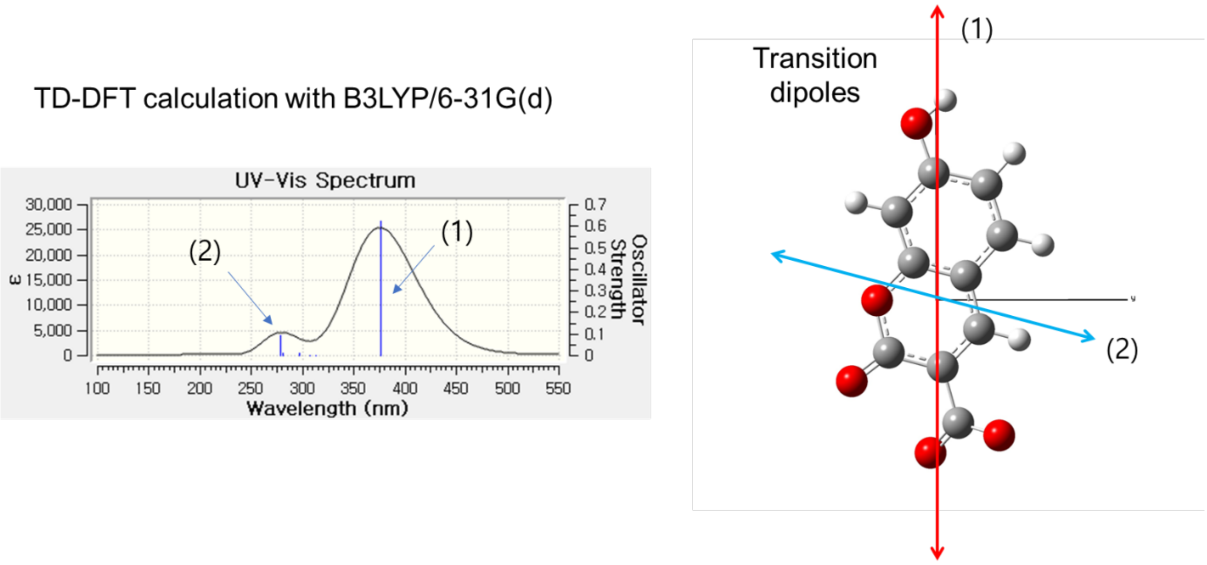

Figure S2. TD-DFT (time-dependent density function theory) calculation with B3LYP/6-31G(d) basis for the deprotonated OHCCA.[4] Simulated absorption and corresponding transition dipoles are represented.

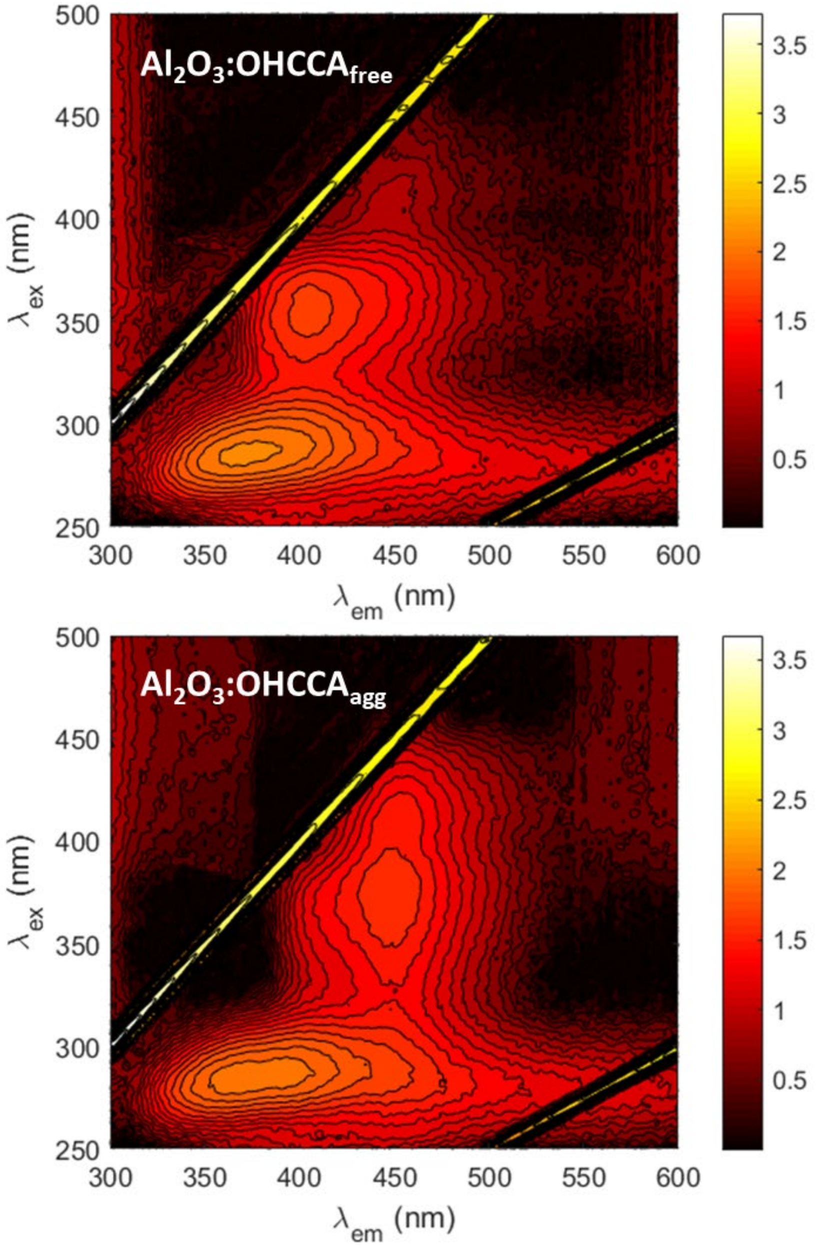

Figure S3. 2D fluorescence spectra of **Al_2O_3:OHCCA$_{free}$ and Al_2O_3:OHCCA$_{agg}$** films, where the strong emission intensity at the excitation of 290 nm is due to the BK7 window.

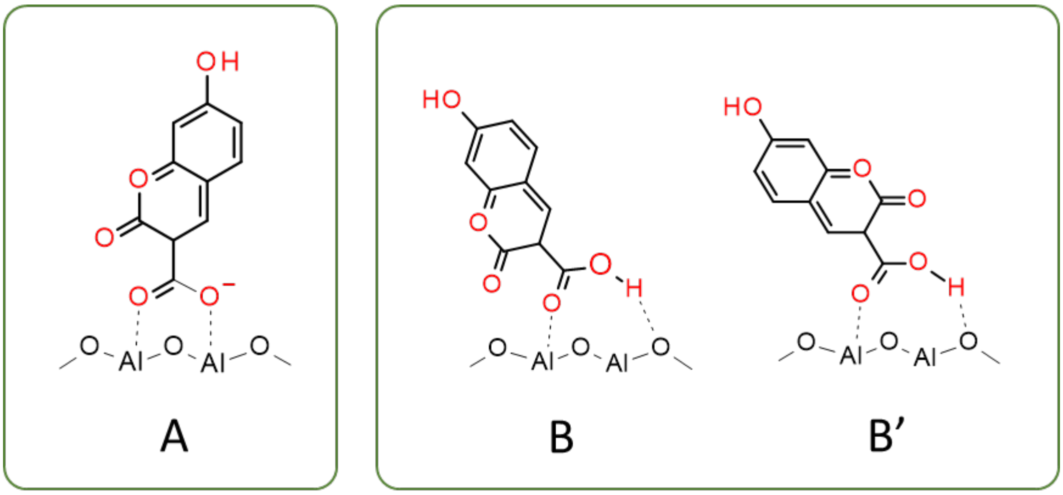

Figure S4. Two representative anchoring modes of OHCCA on the surface of Al_2O_3. A: bidentate binding mode, B and B’: two tilted conformers with the monodentate binding with/without H-bond.

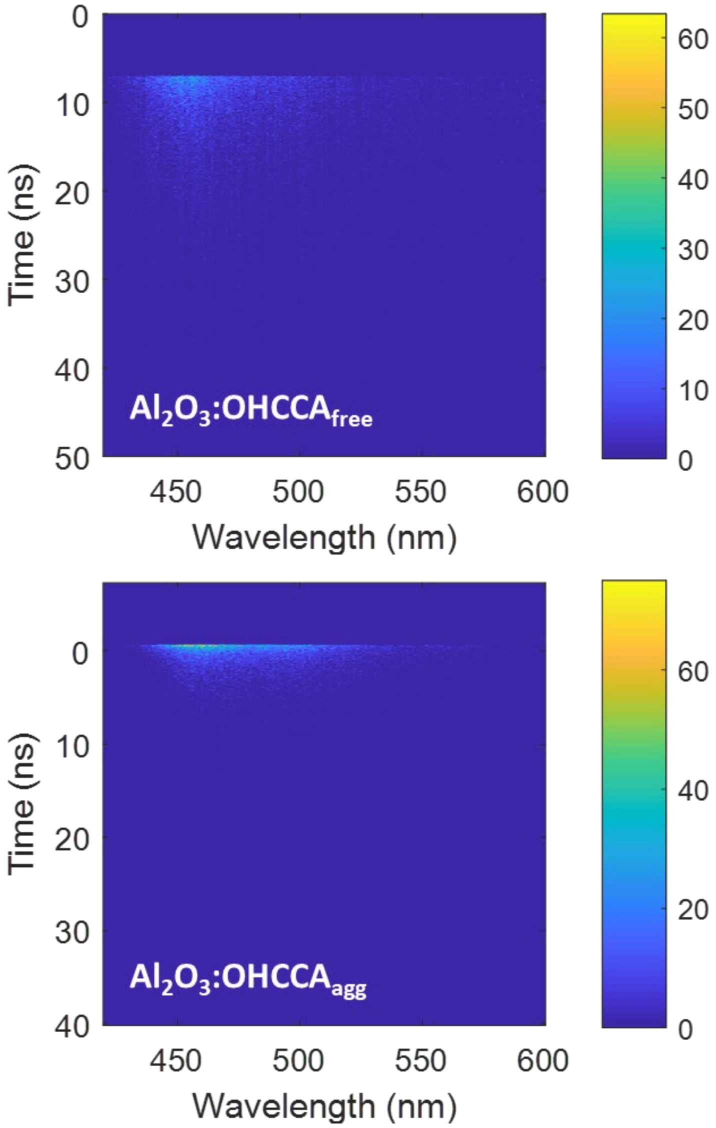

Figure S5. Picosecond TRES images of **Al_2O_3:OHCCA$_{free}$ and Al_2O_3:OHCCA$_{agg}$** films.

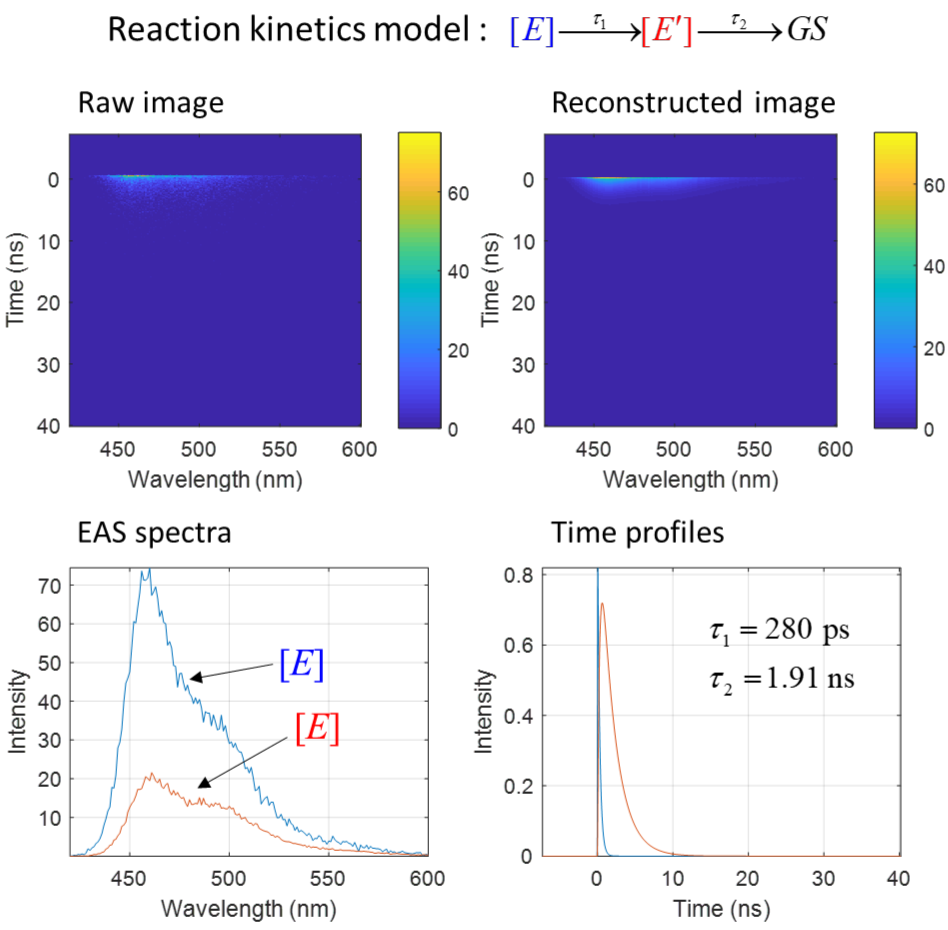

Figure S6. The global target analysis of TRES data for **Al_2O_3:OHCCA$_{agg}$** film: reconstructed image, EAS spectra, and time profiles are from M, F, and D vectors obtained by the global target analysis.

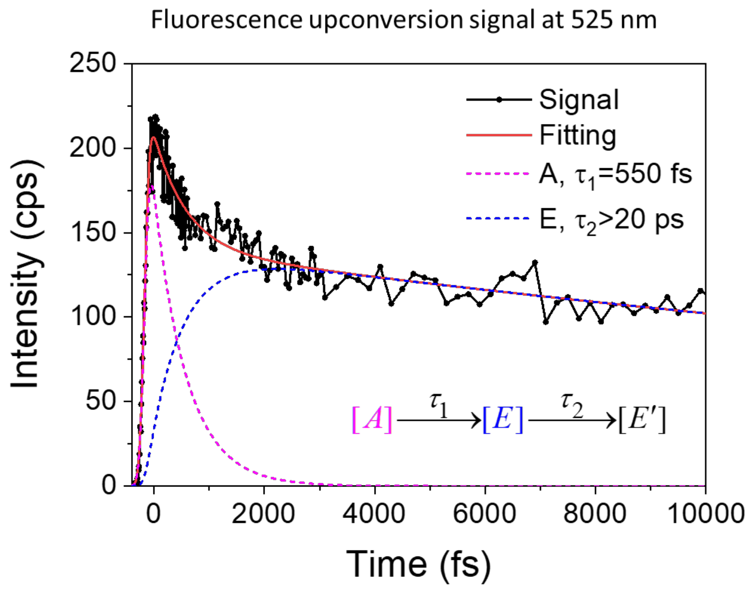

Figure S7. Model populations (dotted lines) and time constants of the first-order kinetic fit to the fluorescence up-conversion signal of **Al_2O_3:OHCCA$_{agg}$**. The detection wavelength was 525 nm.

References

[1] I.H.M. van Stokkum, D.S. Larsen, R. van Grondelle, Global and target analysis of time-resolved spectra, BBA-Bioenergetics 1657 (2004) 82-104.

[2] E.R. Henry, J. Hofrichter, Singular Value Decomposition - Application to Analysis of Experimental-Data, Method Enzymol 210 (1992) 129-192.

[3] B.S. Veldkamp, W.S. Han, S.M. Dyar, S.W. Eaton, M.A. Ratner, M.R. Wasielewski, Photoinitiated multi-step charge separation and ultrafast charge transfer induced dissociation in a pyridyl-linked photosensitizer-cobaloxime assembly, Energ. Environ. Sci. 6 (2013) 1917-1928.

[4] Frisch, M. J.; Trucks, G. W.; Schlegel, H. B.; Scuseria, G. E.; Robb, M. A.; Cheeseman, J. R.; Scalmani, G.; Barone, V.; Petersson, G. A.; Nakatsuji, H.; et al. Gaussian 16W Rev. B.01. **2016**.
